# Author Correction: SARS-CoV-2 infection and COVID-19 vaccination rates in pregnant women in Scotland

**DOI:** 10.1038/s41591-022-01730-5

**Published:** 2022-02-04

**Authors:** Sarah J. Stock, Jade Carruthers, Clara Calvert, Cheryl Denny, Jack Donaghy, Anna Goulding, Lisa E. M. Hopcroft, Leanne Hopkins, Terry McLaughlin, Jiafeng Pan, Ting Shi, Bob Taylor, Utkarsh Agrawal, Bonnie Auyeung, Srinivasa Vittal Katikireddi, Colin McCowan, Josie Murray, Colin R. Simpson, Chris Robertson, Eleftheria Vasileiou, Aziz Sheikh, Rachael Wood

**Affiliations:** 1 University of Edinburgh Usher Institute, Edinburgh, UK; 2 Public Health Scotland, Scotland, UK; 3 The DataLab, Nuffield Department of Primary Care Health Sciences, University of Oxford, Oxford, UK; 4 School of Medicine, University of St Andrews, St Andrews, UK; 5 School of Philosophy, Psychology and Language Sciences, University of Edinburgh, Edinburgh, UK; 6 MRC/CSO Social & Public Health Sciences Unit, University of Glasgow, Glasgow, UK; 7 Institute of Health & Wellbeing, University of Glasgow, Glasgow, UK; 8 School of Health, Wellington Faculty of Health, Victoria University of Wellington, Wellington, New Zealand; 9 Department of Mathematics and Statistics, University of Strathclyde, Glasgow, UK

**Keywords:** Viral infection, Epidemiology, Epidemiology

Correction to: *Nature Medicine* 10.1038/s41591-021-01666-2, published online 13 January 2022.

In the version of this article initially published, a Statistical Analysis subsection was not present in the Methods and has now been included. Further, the Data availability statement has been amended to clarify that patient-level data may be available to approved researchers after securing relevant permissions from Public Health Scotland via the Public Benefit and Privacy Panel.

The changes have been made to the online version of the article.

